# Accuracy of serum procalcitonin for the diagnosis of sepsis in neonates and children with systemic inflammatory syndrome: a meta-analysis

**DOI:** 10.1186/s12879-017-2396-7

**Published:** 2017-04-24

**Authors:** Giuseppe Pontrelli, Franco De Crescenzo, Roberto Buzzetti, Alessandro Jenkner, Sara Balduzzi, Francesca Calò Carducci, Donato Amodio, Maia De Luca, Sara Chiurchiù, Elin Haf Davies, Giorgia Copponi, Alessandra Simonetti, Elena Ferretti, Valeria Di Franco, Virginia Rasi, Martina Della Corte, Luca Gramatica, Marco Ciabattini, Susanna Livadiotti, Paolo Rossi

**Affiliations:** 1 Clinical Trial Unit, University Department of Paediatrics, Bambino Gesù Children’s Hospital, IRCCS, Piazza Sant’ Onofrio 4, 00100 Rome, Italy; 2 Institute of Psychiatry and Psychology, Catholic University of Sacred Heart, Largo Francesco Vito, 1, 00168 Rome, Italy; 3 Immunological and Infectious Disease Unit, University Department of Paediatrics, Bambino Gesù Children’s Hospital, IRCCS, Piazza Sant’ Onofrio 4, 00100 Rome, Italy; 4 Italian Cochrane Centre, Department of Diagnostic, Clinical and Public Health Medicine, University of Modena and Reggio Emilia, Modena, Italy; 5 Paediatric European Network for Treatment of AIDS, Via Giustiniani 3, 35128 Padova, Italy; 6 Department of Anaesthesiology and Intensive Care, Catholic University of Sacred Heart, Largo Francesco Vito, 1, 00168 Rome, Italy; 7 Department of Biomedicine and Prevention, University of Rome “Tor Vergata”, Via Montpellier 1, 00133 Rome, Italy

**Keywords:** Procalcitonin, Sepsis, Systemic inflammatory response syndrome, Meta-analysis, Child, Infant, Biological markers

## Abstract

**Background:**

A number of biomarkers have been studied for the diagnosis of sepsis in paediatrics, but no gold standard has been identified. Procalcitonin (PCT) was demonstrated to be an accurate biomarker for the diagnosis of sepsis in adults and showed to be promising in paediatrics. Our study reviewed the diagnostic accuracy of PCT as an early biomarker of sepsis in neonates and children with suspected sepsis.

**Methods:**

A comprehensive literature search was carried out in Medline/Pubmed, Embase, ISI Web of Science, CINAHL and Cochrane Library, for studies assessing PCT accuracy in the diagnosis of sepsis in children and neonates with suspected sepsis. Studies in which the presence of infection had been confirmed microbiologically or classified as “probable” by chart review were included. Studies comparing patients to healthy subjects were excluded. We analysed data on neonates and children separately.

Our primary outcome was the diagnostic accuracy of PCT at the cut-off of 2-2.5 ng/ml, while as secondary outcomes we analysed PCT cut-offs <2 ng/ml and >2.5 ng/ml. Pooled sensitivities and specificities were calculated by a bivariate meta-analysis and heterogeneity was graphically evaluated.

**Results:**

We included 17 studies, with a total of 1408 patients (1086 neonates and 322 children). Studies on neonates with early onset sepsis (EOS) and late onset sepsis (LOS) were grouped together. In the neonatal group, we calculated a sensitivity of 0.85, confidence interval (CI) (0.76; 0.90) and specificity of 0.54, CI (0.38; 0.70) at the PCT cut-off of 2.0-2.5 ng/ml. In the paediatric group it was not possible to undertake a pooled analysis at the PCT cut-off of 2.0-2.5 ng/ml, due to the paucity of the studies.

**Conclusions:**

PCT shows a moderate accuracy for the diagnosis of sepsis in neonates with suspected sepsis at the cut-off of 2.0-2.5 ng/ml. More studies with high methodological quality are warranted, particularly in neonates, studies considering EOS and LOS separately are needed to improve specificity.

**Trial registration:**

PROSPERO Identifier: CRD42016033809. Registered 30 Jan 2016.

**Electronic supplementary material:**

The online version of this article (doi:10.1186/s12879-017-2396-7) contains supplementary material, which is available to authorized users.

## Background

Sepsis is an on-going clinical problem, and a leading cause of death in adults and children. It has been defined as a systemic inflammatory response syndrome (SIRS) caused by bloodstream infections [1, 2] or, more recently, as life-threatening organ dysfunction caused by a deregulated host response to infection [3].

Several inflammatory mediators are involved in the pathogenesis of sepsis: coagulation, innate and adaptive immune response, intermediary metabolism products, all together interacting and leading to this abnormal response [4].

SIRS may not only be determined by infections but also non-infectious causes, such as autoimmune disorders, pancreatitis, vasculitis, thromboembolism, burns, or surgery procedures. SIRS in paediatrics is defined by at least two of the following parameters, one of which must be abnormal temperature or leukocyte count: hyperthermia or hypothermia (>38.5 °C or <36 °C), tachycardia (defined as a mean heart rate more than two standard deviations above normal for age) or bradycardia for children less than 1 year old (defined as a mean heart rate < 10th percentile for age), tachypnea (mean respiratory rate more than two standard deviations above normal for age), leukocyte count elevated or depressed for age, or >10% immature neutrophils [5].

Sepsis has been categorized in the neonatal period as early onset sepsis (EOS) and late onset sepsis (LOS) if occurring in the first 72 h after birth or later. The main risk factors and the pathogens associated are different, being in EOS chorioamnionitis, bacterial colonization of the birth canal, Group B streptococci (GBS) and *Escherichia coli*; in LOS healthcare acquired infections, preterm delivery and Coagulase negative streptococci [6].

The diagnosis of sepsis is made in children with SIRS in presence of a proven infection by a positive blood culture, or probable infection by a complete and often a posteriori review of clinical, laboratory and radiological data [2, 4, 5]. Blood culture is currently the reference standard for the confirmation of the diagnosis of sepsis. However, even if it represents a fundamental tool for the etiologic diagnosis and for the establishment of a targeted therapy, it has important and significant limitations, such as the time delay in obtaining results and a high percentage of false negatives [7, 8]. Delay in antibiotic treatment of infected children is associated with a significant risk in terms of mortality and morbidity [6].

Biomarkers can play an important role in providing a timely diagnosis of sepsis, helping the differential diagnosis with non-infectious SIRS and the decision-making in the initial management. In paediatrics, the most frequently employed biomarker to differentiate sepsis from non-infectious SIRS is the C-reactive protein (CRP), which, however, is highly non-specific and has an unfavourable kinetics [9]. Among the different molecules investigated as biomarkers of sepsis, procalcitonin (PCT) seems to be one of the most promising [10–12]. PCT is a 116-aminoacids pro-hormone, physiologically produced by thyroid C-cells as precursor of calcitonin, an acute phase protein secreted by several tissues in response to various endogenous and exogenous stimuli such as cytokines and lipopolysaccharide, acting as a chemo-attractant factor on blood monocytes [13]. In healthy neonates, plasma PCT values increase gradually after birth, reach peak values after 24 h of age (mean 1.5-2.5 ng/ml, range 0.1-20 ng/ml) and then decrease to normal values below 0.5 ng/ml by 48-72 h of age. A number of studies in children and neonates after 72 h of age, demonstrated that PCT values less than 0.5 ng/ml seem to be normal; increases to 0.5-2 ng/ml seem to be related to non-infectious inflammation, viral or focal bacterial infections; increases above a PCT value of 2-2.5 ng/ml, seem to be related to bacterial or fungal systemic infections [14–16]. PCT as a diagnostic biomarker for sepsis in individuals with SIRS has been well evaluated in adults [11, 17–25]. Various meta-analyses in paediatric age groups have been done so far [16, 26, 27], but no one has evaluated the role of PCT in sepsis for children and neonates with SIRS or suspected sepsis, the most useful setting for clinicians. Therefore, our objective was to assess the diagnostic accuracy of PCT to differentiate between sepsis and systematic inflammatory response syndromes of non-infectious origin in children and neonates with suspected sepsis.

## Methods

The protocol for this review was accepted and registered on PROSPERO international prospective register of systematic reviews under the number CRD42016033809.

### Search strategy

We searched Medline/Pubmed, Embase, ISI Web of Science, CINAHL, Cochrane Library, for studies that assessed the accuracy of PCT for the diagnosis of sepsis in neonates and in children over 44 weeks, defined as “paediatric age”. The search algorithm used for each database is available in the Additional file 1. No start date limit was used and the search strategy was performed in August 2014 and updated until the cut off date of December 2015. To expand our search, reference lists of the retrieved articles were also screened for additional studies. We also searched grey literature through Open Sigle. No language limits were applied.

### Selection criteria

We included all studies regardless of study design: prospective or retrospective that met the following criteria: assessing the accuracy of PCT for diagnosis of sepsis in children and neonates with SIRS or suspected sepsis, providing sensitivity (true-positive rate) and specificity (true-negative rate). The presence of infection had to be microbiologically confirmed (positive culture, microscopy or polymerase chain reaction) or evaluated as probable by chart review.

We excluded: a) articles not regarding sepsis or not assessing PCT; b) studies not in children or neonates; c) studies using only healthy children or neonates as controls; d) studies on children or neonates without probable infection; e) review articles, editorials or letters, expert opinions, comments and animal experiments.

At least two reviewers independently evaluated titles and abstracts and selected relevant studies for inclusion. If this could not be done reliably by title and abstract, the full text version was retrieved. Any disagreement was resolved by discussion within reviewers, or by an independent reviewer. Reason for exclusion of studies was recorded.

### Data extraction

Data about the following variables were extracted independently by at least two reviewers: year of publication, clinical setting (neonatal or paediatric intensive care unit, or general ward), age at diagnosis, sample size, design of the study, prevalence of sepsis, laboratory methods, cut-off points, timing of tests, inclusion criteria (SIRS or suspected sepsis), outcome diagnosis (sepsis confirmed by microbiological test or by chart review) and when reported, the main measures of test accuracy. Any disagreement on data extraction was resolved by consensus.

### Quality assessment

The methodological quality of each study was assessed using a checklist based on criteria adapted from the Cochrane Collaboration guidelines and the Quality Assessment Tool for Diagnostic Accuracy Studies (QUADAS-2 score) and applied to each included study. QUADAS-2 is made of four domains: patient selection, index test, reference standard, flow and timing. Each domain evaluates the risk of bias and for the first three there is also an assessment of applicability. Signalling questions are included to help in the judgement about risk of bias [28]. Each question was assessed by scoring it as “yes”, “no”, or “unclear” and the risks of bias and the concerns on applicability were scored as “high”, “low” or “unclear”, depending on the answers of the signalling questions. At least two authors scored independently, and differences were resolved by consensus or by a third reviewer.

### Statistical analysis

We extracted information on true positives (tp), false negatives (fn), false positives (fp), and true negatives (tn) for each study. We carried out primary analyses considering neonates and children as two different groups. We then conducted further analyses on neonates with suspected EOS as a stand-alone group and on neonates with suspected LOS and children grouped together. We used two-by-two data in order to calculate sensitivities and specificities, along with their 95% confidence intervals. They were graphically evaluated by using forest plots and by plotting study results in ROC space.

Studies were divided into subgroups depending on the specific PCT cut-off considered in their test accuracy analyses. We grouped the studies according to three different PCT ranges: <2 ng/L; 2-2,5 ng/L; >2,5 ng/L on the basis of preliminary observational studies and PCT nomograms [14, 29]. These nomograms showed differences between neonatal and paediatric cut-off values of PCT in healthy populations. Moreover, the 2-2.5 ng/ml cut-off was chosen as proposed by the Expert Meeting on Neonatal and Paediatric Sepsis of European Medicines Agency [30]. If a study reported results at different cut-offs, we chose one of them for each subgroup.

If adequate data were available, meta-analyses were performed by using the bivariate model [31] to produce summary sensitivities and specificities. A random-effects model jointly synthesizes sensitivity and specificity by allowing for correlation between them across studies. Average operating points, along with their confidence and predictive regions for each subgroup were calculated whenever possible (i.e. they were not calculated if there were less than three studies in a subgroup). Heterogeneity was graphically evaluated [32]; where heterogeneity was high, the 95% prediction region was much larger than the 95% confidence region. All analyses were performed using Review Manager [33] and STATA 13 software [34].

## Results

### Selected studies

**Fig. 1:**
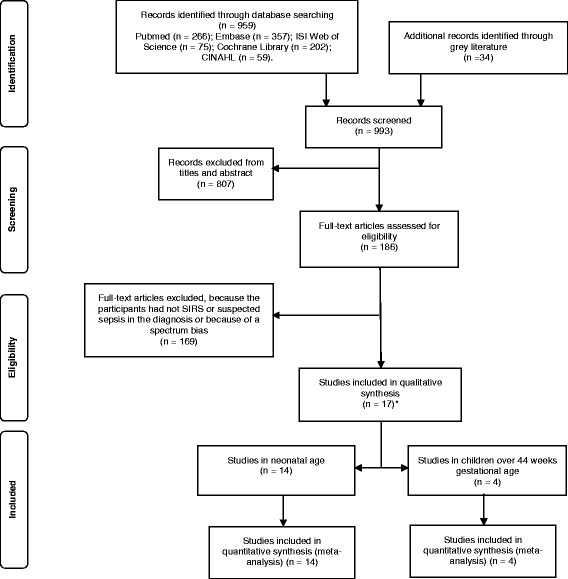
Preferred reporting items for systematic reviews and meta-analyses (PRISMA) flow chart. Literature search and selection. *One study assessed both neonates and children over 44 weeks of gestational age

The literature search generated 993 articles*.* After reviewing the titles and abstracts, we excluded 807 studies, because they were either reviews or studies in adults or not focused on sepsis or using only healthy subjects as controls. A total of 186 studies were retrieved in full text and assessed for eligibility. Of these 169 were excluded due to their poor design and/or because of identified biases, mostly spectrum bias, or because SIRS was not among the inclusion criteria. Indeed, many studies have been focusing on PCT for the diagnosis of serious bacterial infections such as pneumonia, meningitis or pyelonephritis, but not SIRS and sepsis. In total, 17 studies comprising 1408 patients (1086 neonates and 322 children) were included in the qualitative and quantitative analysis. Of these, 13 studies were in neonates, 3 studies were in children, while one study included both [35]. The selection of the studies has been summarized in the flow chart (see Fig. 1).

### Study characteristics

We split the studies in two groups according to age: neonatal [35–48] and paediatric [35, 49–51]. Moreover, we stratified the data in subgroups according to the cut-off considered in the primary study. PCT cut-offs varied, ranging from 0.5 ng/ml [39, 43] to 25 ng/ml [37] in the neonatal group, and from 0.28 ng/ml [35] to 10 ng/ml [52] in the paediatric group. The study design was prospective for both neonatal and paediatric age, with the only exception of a retrospective cross-sectional study [45] in the neonatal age group. The setting of the studies was uniform, being mostly neonatal or paediatric intensive care units, with the exception of four studies [41, 45, 49, 51] in which patients were treated in hospital wards. Most of the studies (15 out of 17) used a qualitative semi-manual PCT assay. The characteristics of the included studies considering neonates and older children are presented respectively in Table 1 and in Table 2. The judgments on the methodological quality of the included studies according to the QUADAS-2 assessments [28] are presented in the Additional file 2.

**Table 1:** Table of included neonatal studies

Study	Design	Age	Early or Late Onset	Setting	n	Prevalence of sepsis (%)	Procalcitonin lab assay	Timing of test	PCT Cut-off (ng/ml)	Inclusion Criteria	Sepsis Diagnosis	Sensitivity	Specificity	AUC ROC
Adib 2012 [36]	Cross-sectional	0 to 28 days	Early and Late	NICU	69	29	PCT-LIA	0 h	1.15	Suspected sepsis	MC	70	40	-
Bender 2008 [37]	Prospective	0-72 h	Early	NICU	123	24	PCT-LIA	0 h	5.75	Suspected sepsis	MC or CR	68	67	-
									25			21	92	-
Bonac 2000 [38]	Prospective	2.5 days	Early	NICU	58	15	PCT-LIA	0 h	9,98	Suspected sepsis	MC or CR	59	82	0.61
								24 h	13.03			50	100	0.73
								48 h	3.07			52	91	-
Boo 2008 [39]	Prospective	Preterm- 54 days	Early and Late	NICU	87	21	Semi-quantitative PCT-Q	0 h	0,5	Suspected Sepsis	MC	89	41	-
								12-24 h	2			89	65	-
								36-48 h	10			72	75	-
Groselj-Grenc 2009 [35]	Prospective	1-18 days	Early and Late	NICU	46	63	PCT-LIA	0 h	2.28	SIRS	MC or CR	82	48	0.67
								24 h	5.55			31	100	0.64
Guibourdenche 2002 [40]	Prospective	1 day	Early	NICU	120	18	PCT-LIA	0-72 h	2.5	Suspected sepsis	MC or CR	87	90	-
Koskenvuo 2003 [41]	Prospective	0-72 h	Early	Medical	22	22	PCT-LIA	12 h	2	SIRS	MC or CR	86.4	47.2	-
Lopez Sastre 2006 [42]	Prospective	4 - 28 days of life	Late	NICU	100	61	PCT-LIA	0 h	0.59	Suspected sepsis	MC	82	81	0.78
								12-24 h	1.34			74	81	0.81
								36-48 h	0.69			87	73	0.80
Naher 2011 [43]	Cross sectional	37wks gestational age	Early and Late	NICU	50	80	Semi-quantitative PCT-Q	0 h	0.5	Suspected Sepsis	MC or CR	70	90	0.77
Resch 2003 [44]	Prospective	35.5 weeks Gestational age	Early	NICU	68	61	PCT-LIA	0 h	2	Suspected Sepsis	MC or CR	83	61	-
									6			77	91	-
									14			63	100	-
Sakha 2008 [45]	Cross-sectional	0 to 28 days	Early and Late	Neonatal Ward	117	23	PCT-LIA	-	2.5	Suspected sepsis	MC	67	50	0.61
Schlapbach 2013 [46]	Prospective	0-72 h of life, gestational age > 34 weeks	Early	NICU	137	24.1	PCT-LIA	0 h	2	Suspected sepsis	MC or CR	87,9	51	-
Vazzalwar 2005 [47]	Prospective	≥ 7 days	Late	NICU	51	35.3	PCT-LIA	0 h	0.5	Suspected sepsis	MC or CR	94.4	36.4	-
									1			77.8	63.6	-
Zahedpasha 2009 [48]	Prospective	0 to 28 days	Early and Late	NICU	38	28.9	PCT-LIA	0 h	0,5	Suspected sepsis	MC or CR	100	25.9	-
									2			100	33.3	-
									10			90.9	85.2	-

*AUC* Area Under the Curve, *CR* Chart Review, *MC* Microbiologically Confirmed, *NICU* Neonatal Intensive-Care Unit, *PCT* Procalcitonin, *PCT LIA* Procalcitonin Manual Assay, *PCT-Q* Procalcitonin Rapid Assay, *ROC* Receiver Operating Characteristic, *SIRS* Systemic Inflammatory Response Syndrome

**Table 2:** Table of included paediatric studies

Study	Design	Age	Setting	n	Prevalence of sepsis	Procalcitonin lab assay	Timing of test	PCT Cut-off(ng/ml)	Inclusion Criteria	Outcome Diagnosis	Sensitivity %	Specificity %	AUC ROC
Calò Carducci 2014 [49]	Prospective	2 months	Hospital Ward	64	64	PCT-LIA	0 h	0.55	SIRS	MC or CR	88	74	-
Groselj-Grenc 2009 [35]	Prospective	1 month-12 years	PICU	36	67	PCT-LIA	0 h	0.28	SIRS	CR	83	75	0.83
							24 h	0.65			81	88	0.86
Pourakbari 2010 [51]	Prospective	42.8 +/− 3,5 months	Hospital ward + Emergency Dept	158	79	PCT-Q	0 h	0.5	SIRS	MC	65	47	-
								2			44	80	-
								10			30	89	-
Simon 2008 [50]	Prospective	80 months +/− 71	PICU	64	39	PCT-LIA	24 h	0.5	SIRS	CR	82	36	0.71
								2.5			68	74	
								5			50	82	

*AUC* Area Under the Curve, *CR* Chart Review, *MC* Microbiologically Confirmed, *NICU* Neonatal Intensive-Care Unit, *PCT* Procalcitonin, *PCT LIA* Procalcitonin Manual Assay, *PCT-Q* Procalcitonin Rapid Assay, *ROC* Receiver Operating Characteristic, *SIRS* Systemic Inflammatory Response Syndrome

### Data synthesis in neonatal age

**Fig. 2:**
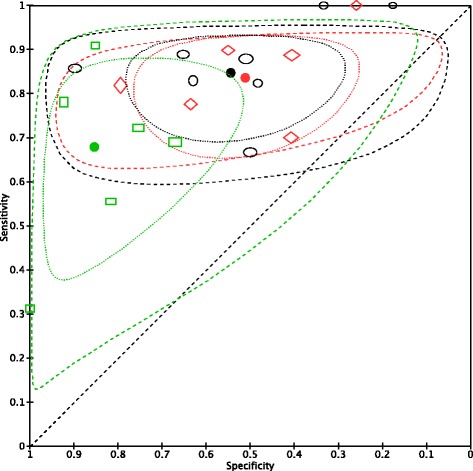
Representation in the ROC space of neonatal studies. Representation in the ROC space of studies on PCT for diagnosis of sepsis in neonatal age, divided by cut-off subgroup, and summary sensitivity and specificity points along with their 95% confidence and prediction regions. (ROC, receiver operating characteristic). Legend:  PCT neon – cut-off <2  PCT neon – cut-off > 2.5  PCT neon – cut-off =2/2.5

**Fig. 3:**
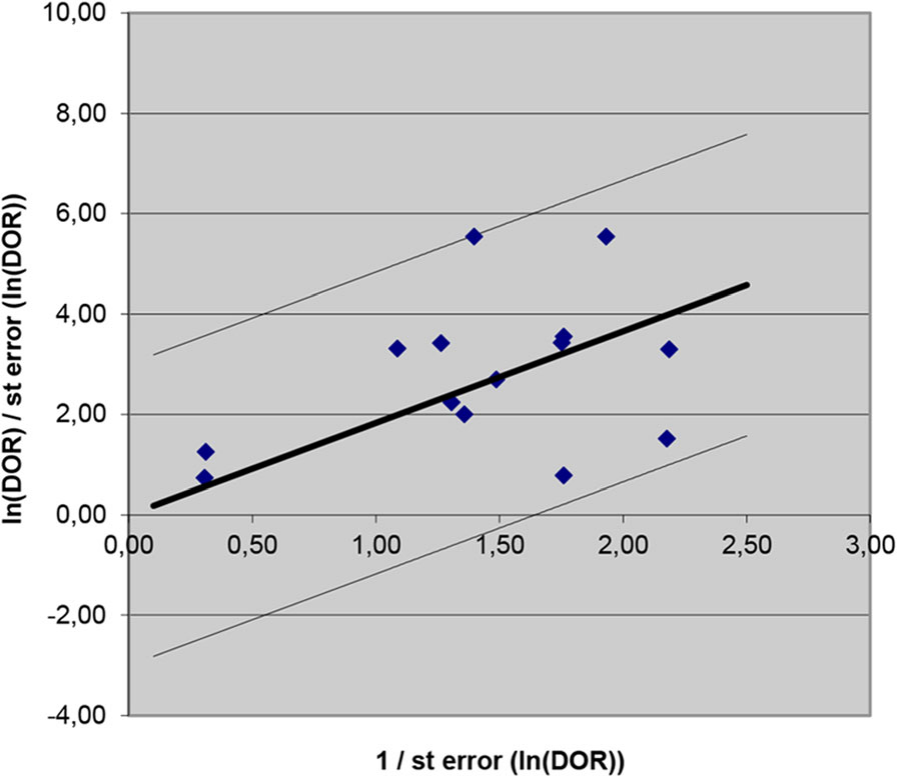
Galbraith plot. Heterogeneity of selected neonatal studies. Galbraith plot for neonatal studies. The standardised lnDOR = lnDOR/se was plotted (y-axis) against the inverse of the se (1/se) (x-axis). A regression line going through the origin was calculated, together with 95% boundaries (starting at +2 and −2 on the y-axis). (DOR, diagnostic odds ratio)

Meta-analytic results show that when using a PCT cut-off of between 2.0 and 2.5 ng/ml, pooled sensitivity is 0.85 (95% CI 0.76; 0.90) and pooled specificity is 0.54 (95% CI 0.38; 0.70) (see Table 3). Figure 2 shows study results in neonates plotted in the ROC space, broken down by cut-off subgroups with the 95% confidence interval and predictive regions for the summary sensitivity and specificity. The data extracted for the analysis are presented extensively in the Additional file 3. The forest plot for neonatal age is presented in the Additional file 4. When using a PCT cut-off of <2.0 ng/ml, pooled sensitivity is 0.84 (95% CI 0.75; 0.90) and pooled specificity is 0.51 (95% CI 0.37; 0.65). With a PCT cut-off of >2.5 ng/ml, pooled sensitivity is 0.68 (95% CI 0.52; 0.80) and pooled specificity is 0.85 (95% CI 0.70; 0.93). The Galbraith plot does not show heterogeneity among the results (see the Fig. 3). Plotting the studies divided by cut-off subgroups and neonatal EOS or LOS in the ROC suggests that PCT accuracy changes considerably depending on the onset: neonates with EOS need a higher PCT cut-off, while neonates with LOS require a lower PCT cut-off (see Additional file 5).

**Table 3:** Summary statistics of Procalcitonin for diagnosis of sepsis in neonatal age according the age of onset, and cut-off used in the studies

		Onset		Summary statistics
Cut-off	Early	Early/Late	Late	
<2		Boo 2008 (0.5) Naher 2011 (0.5) Zahedpasha 2009 (0.5) Adib 2012 (1.15)	Lopez Sastre 2006 (0.59) Vazzalwar 2004 (1)	SE = 0.84 (0.75; 0.90) SP = 0.51 (0.37; 0.65)
2-2.5	Resch 2003 (2) Guibourdenche 2002 (2.5)	Boo 2008 (2) Zahedpasha 2009 (2) Groselj-Grenc 2009 (2.28) Sakha 2008 (2.5) Koskenuovo 2003 (2) Schlapbach 2013 (2)		SE = 0.85 (0.76; 0.90) SP = 0.54 (0.38; 0.70)
>2.5	Bender 2008 (5.75) Resch 2003 (6) Bonac 2000 (9.98)	Boo 2008 (10) Groselj-Grenc 2009 (5.55) Zahedpasha 2009 (10)		SE = 0.68 (0.52; 0.80) SP = 0.85 (0.70; 0.93)

*SE* Sensitivity, *SP* Specificity, Actual cut-off reported in the brackets

The additional analyses performed on the four studies which included only neonates with suspected EOS at a PCT cut-off of ≥2.5 ng/ml identified a pooled sensitivity of 0.75 (95% CI 0.64; 0.84), and a pooled specificity of 0.83 (95% CI 0.71; 0.91) (see Additional files 6 and 7).

### Data synthesis in paediatric age

**Fig. 4:**
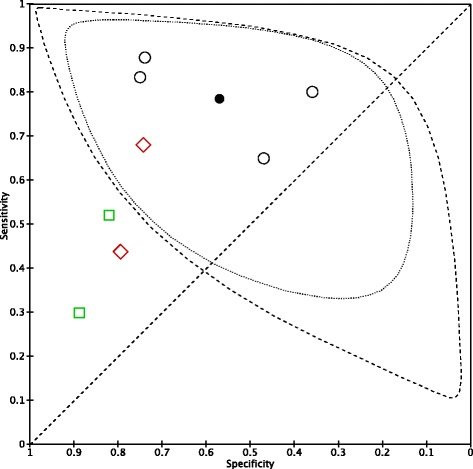
Representation in the ROC space of paediatric studies. Representation in the ROC space of the studies of PCT for diagnosis of sepsis in paediatric age, divided by cut-off subgroup, and summary sensitivity and specificity points along with their 95% confidence and prediction regions. (ROC, receiver operating characteristic). Legend:  PCT paed – cut-off <2  PCT paed – cut-off > 2.5  PCT paed – cut-off =2/2.5

**Fig. 5:**
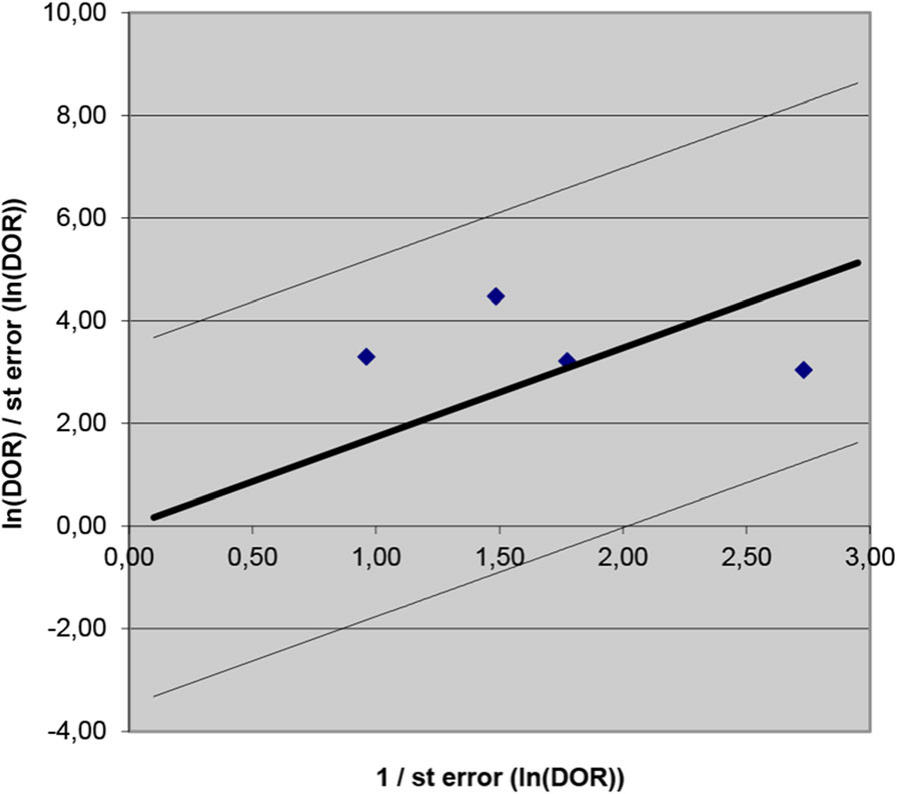
Galbraith plot. Heterogeneity of selected paediatric studies. Galbraith plot for paediatric studies. The standardised lnDOR = lnDOR/se was plotted (y-axis) against the inverse of the se (1/se) (x-axis). A regression line going through the origin was calculated, together with 95% boundaries (starting at +2 and −2 on the y-axis). (DOR, diagnostic odds ratio)

In the older patient group, it was only possible to meta-analyse studies using a PCT cut-off of <2.0 ng/ml: the pooled sensitivity was 0.78 (95% CI 0.66; 0.87) and the pooled specificity of 0.57 (95% CI 0.40; 0.73). Figure 4 shows the study results in the paediatric age in the ROC space, broken down by cut-off subgroups, along with the 95% confidence and predictive regions for pooled sensitivity and specificity. The Galbraith plot does not show heterogeneity among the results (see the Fig. 5). The data extracted for the analysis are presented extensively in the Additional file 8. The forest plot for paediatric age is presented in the Additional file 9. Grouping neonates with suspected LOS and paediatric patients together allows for the assessment of PCT accuracy at the only cut-off of <2 ng/ml: the pooled sensitivity was 0.79 (95% CI 0.71; 0.85); the pooled specificity was 0.63 (95% CI 0.48; 0.75) (see the Additional file 10 and the Additional file 11).

## Discussion

This is the first systematic review assessing PCT accuracy for sepsis in neonates and children with suspected sepsis or SIRS excluding healthy subjects, and therefore providing more useful information for clinicians. According to our meta-analysis, PCT at the cut-off of 2-2.5 ng/ml shows the best sensitivity and moderate accuracy for the diagnosis of sepsis in neonates with suspected sepsis. The sensitivity is high, but associated with a low specificity. Even if suboptimal, this could be considered acceptable, because of the high mortality rate of the condition, and the need to maintain a low false negative ratio.

In neonates, the PCT cut-off of <2 ng/ml shows a high sensitivity with a low specificity, similar to those of cut-off of 2-2.5 ng/mL; the PCT cut-off of >2.5 ng/ml shows lower, unacceptable values for sensitivity, and higher specificity. It is noteworthy to consider that studies on EOS with a PCT cut-off <2 ng/ml were all grouped together with LOS, and the two studies focusing on LOS only [42, 47] found that the PCT at this cut-off had a better accuracy, higher specificity and similar sensitivity, if compared with studies grouping EOS and LOS. This suggests that the use of two different cut-offs could improve accuracy in these two populations: PCT cut-off could be higher for neonates with EOS than for neonates with LOS. This is consistent with existing studies on healthy neonates [14, 29]. It is important that in future studies on EOS the diagnostic value of age-adjusted PCT cut-offs will be assessed in association with other serum biomarkers [46, 53].

Unfortunately, at present, there are not enough studies to perform a meta-analysis on the diagnostic accuracy of PCT at the cut-off of 2-2.5 ng/ml in older children with suspected sepsis or SIRS. In this population, the results on the PCT cut off of <2.0 ng/ml show an overall moderate accuracy, but the limited sample size and quality of the studies included indicate that further studies are needed and no clinical recommendation is possible at this stage. Additional analyses that considered the few studies only on EOS, and grouped together LOS and paediatric patients cannot overcome this limitation.

The study showed limitations linked to the current methodology of primary sepsis research, such as the non-uniform definition of sepsis: some studies considered sepsis only if confirmed by positive blood culture, microscopy or polymerase chain reaction (microbiologically confirmed) while others considered also “probable sepsis”, after a complete review of the patient chart with assessment of clinical, radiological and laboratory data. We included studied that considered both microbiologically confirmed and probable sepsis, but they did not provide detailed information about how the infection was confirmed. In addition, blood samples were drawn without a precise timing (i.e. “at time of admission” or “before antibiotic therapy”, which do not necessarily coincide). Furthermore, the scarcity of studies differentiating EOS and LOS in neonates hampered a more specific and informative analysis.

In 2016, a new definition of sepsis attributing a primary role to organ dysfunction was proposed in adults, aiming to offer greater consistency for epidemiological studies and clinical trials [3]. In order to improve diagnosis and decrease the mortality of sepsis in neonatal and paediatric population, we need soon additional studies of high methodological quality, accounting the specificity of pathophysiology and age dependent variables.

## Conclusions

In conclusion, in this study we show that PCT has an overall moderate accuracy for the diagnosis of sepsis in neonates with suspected sepsis at the cut-off of 2.0-2.5 ng/ml.

In order to deepen our scientific knowledge on the role of PCT in the diagnosis of neonatal and paediatric sepsis, larger, high quality studies are necessary. More specifically, we need studies responding to the Standards for Reporting of Diagnostic Accuracy (STARD) guidelines, with a previously published and registered protocol, and an adequate sample size. It would also be of paramount importance to include suspected sepsis and exclude healthy subjects, in order to provide more useful information for the clinicians, and be clearly able to differentiate neonates with EOS and LOS. An updated definition of sepsis for paediatric population, similarly to that one proposed for adults, which considers the different pathophysiology and age dependent variables and overrule the current heterogeneity is warranted.

## Key messages

1. PCT shows a moderate diagnostic accuracy at the cut-off of 2-2.5 ng/ml for the diagnosis of sepsis in neonates with SIRS or suspected sepsis.

2. In neonates, the PCT values should be critically evaluated differentiating EOS and LOS.

3. Further studies with better methodological quality in older children with suspected sepsis evaluating the PCT cut-off of 2-2.5 ng/ml are warranted.

## Additional files

Additional file 1: Search strategy. (PDF 54 kb)

Additional file 2: Table of bias. Table of bias of neonatal and paediatric studies according to QUADAS-2. (PDF 84 kb)

Additional file 3: Table of included neonatal studies. CR, Chart review; DOR, diagnostic odds ratio; LR +, positive likelihood ratio; LR -, negative likelihood ratio; MC, Microbiologically confirmation; PCT, procalcitonin; SIRS, systemic inflammatory response syndrome; MC (Microbiologically confirmation). (PDF 130 kb)

Additional file 4: Forest plot of studies on PCT for diagnosis of sepsis in neonatal age. The forest plot represents in each study the sensitivity and the specificity of PCT, together with the 95% CI for diagnosis of sepsis in neonatal age stratified according cut-off subgroup. (CI, confidence interval; FP, false positive; FN, false negative; PCT, procalcitonin; TP, true positive; TN, true negative). (PDF 743 kb)

Additional file 5: Representation in the ROC space of neonatal studies divided in EOS and LOS. Representation in the ROC space of studies on PCT for diagnosis of sepsis in neonatal age, divided by cut-off subgroup and EOS/LOS. (ROC, receiver operating characteristic). (PDF 46 kb)

Additional file 6: Forest plot and summary statistics of studies on PCT for diagnosis of EOS. The forest plot represents in each study the sensitivity and the specificity of PCT, together with the 95% CI for diagnosis of EOS. (CI, confidence interval; FP, false positive; FN, false negative; PCT, procalcitonin; TP, true positive; TN, true negative). (PDF 323 kb)

Additional file 7: Representation in the ROC space of studies in EOS. Representation in the ROC space of studies on PCT for diagnosis of EOS. (ROC, receiver operating characteristic). (PDF 157 kb)

Additional file 8: Table of included paediatric studies. (DOR, diagnostic odds ratio; LR+, positive likelihood ratio; LR-, negative likelihood ratio; PCT, procalcitonin; SIRS, systemic inflammatory response syndrome; MC, Microbiologically confirmation; CR Chart Review). (PDF 19 kb)

Additional file 9: Forest plot of studies on PCT for diagnosis of sepsis in paediatric age. The forest plot represents in each study the sensitivity and the specificity of PCT, together with the 95% CI for diagnosis of sepsis in paediatric age stratified according cut-off subgroup. (PDF 377 kb)

Additional file 10: Forest plot and summary statistics of studies on PCT for diagnosis of LOS and paediatric sepsis. The forest plot represents in each study the sensitivity and the specificity of PCT, together with the 95% CI for diagnosis of LOS and paediatric sepsis. (CI, confidence interval; FP, false positive; FN, false negative; PCT, procalcitonin; TP, true positive; TN, true negative). (PDF 337 kb)

Additional file 11: Representation in the ROC space of studies in LOS and paediatric sepsis. Representation in the ROC space of studies on PCT for diagnosis in LOS and paediatric sepsis. (PDF 154 kb)

## Abbreviations

AUC: Area under the curve
CI: Confidence interval
CR: Chart review
CRP: C-reactive protein
EOS: Early onset sepsis
FN: False negative
FP: False positive
LOS: Late onset sepsis
MC: Microbiologically confirmation
NICU: Neonatal intensive-care unit
NPV: Negative predictive value
PCT LIA: Procalcitonin immunoluminometric assay
PCT: Procalcitonin
PCT-Q: procalcitonin semiquantitative rapid assay
PPV: Positive predictive value
QUADAS score: Quality Assessment Tool for Diagnostic Accuracy Studies
ROC: Receiver operating characteristic
SIRS: Systemic inflammatory response syndrome
STARD: Standard for Reporting of Diagnostic Accuracy
TN: True negative
TP: True positive
