# Supplementary material for: Accuracy of serum procalcitonin for the diagnosis of sepsis in neonates and children with systemic inflammatory syndrome: a meta-analysis
Source: BMC Infect Dis. 2017 Apr 24;17:302. doi: 10.1186/s12879-017-2396-7 (PMC5404674; doi:10.1186/s12879-017-2396-7)
Supplement: Supplementary file 1 — Search strategy. (PDF 54 kb) [file 12879_2017_2396_MOESM1_ESM.pdf]

Appendix 1. Search strategy.

Search strategy: Embase

1. ‘procalcitonin:.mp‘
2. ‘pct:.mp’
3. 1 OR 2
4. ‘neonat:.mp’
5. ‘newborn:.mp’
6. ‘infant:mp’
7. 4 OR 5 OR 6
8. 3 AND 7

Search strategy: Cochrane Library

1. exp ‘calcitonin’
2. ‘procalcitonin’
3. ‘pct’
4. “calcitonin precursor polyprotein”
5. “CALCA protein, human”
6. “calcitonin/calcitonin-related polypeptide, alpha protein, human”
7. “CGRP1 protein, human”
8. “CALC1 protein, human”
9. 1 OR 2 OR 3 OR 4 OR 5 OR 6 OR 7 OR 8
10. ‘neonat*’
11. ‘newborn*’
12. ‘infant*’
13. “Infants, Newborn”
14. “Newborn Infant”
15. “ Newborn Infants”
16. 10 OR 11 OR 12 OR 13 OR 14 OR 15
17. 9 AND 16

Search strategy: Medline-Pubmed

1. exp ‘calcitonin’/
2. ‘procalcitonin’.mp
3. ‘pct’.mp
4. “calcitonin precursor polyprotein”
5. “CALCA protein, human”
6. “calcitonin/calcitonin-related polypeptide, alpha protein, human”
7. “CGRP1 protein, human”
8. “CALC1 protein, human”
9. 1 OR 2 OR 3 OR 4 OR 5 OR 6 OR 7 OR 8
10. exp. “Infants, Newborn”/
11. ‘newborn*’.mp
12. ‘infant*’.mp
13. ‘neonat*’.mp
14. “Newborn Infant”
15. “Newborn Infants”
16. 10 OR 11 OR 12 OR 13 OR 14 OR 15
17. 9 AND 16

Search strategy: CINHAL

1. (MH "procalcitonin+")
2. "procalcitonin"
3. "pct"
4. "calcitonin precursor polyprotein"
5. 1 OR 2 OR 3 OR 4
6. "newborn"
7. "infants"
8. "children"
9. 6 OR 7 OR 8
10. 5 AND 9

Search strategy: Isi Web of Science

1. "calcitonin"
2. procalcitonin.mp
3. pct.mp
4. "calcitonin precursor polyprotein".mp
5. "CALCA protein, human".mp
6. "calcitonin/calcitonin-related polypeptide, alpha protein, human".mp
7. "CGRP1 protein, human".mp
8. "CALC1 protein, human".mp
9. 1 OR 2 OR 3 OR 4 OR 5 OR 6 OR 7 OR 8
10. "Infants, Newborn"
11. newborn*.mp
12. infant*.mp
13. neonat*.mp
14. "Newborn Infant".mp
15. "Newborn Infants".mp
16. 10 OR 11 OR 12 OR 13 OR 14 OR 15
17. 9 AND 16
