# Supplementary material for: Accuracy of serum procalcitonin for the diagnosis of sepsis in neonates and children with systemic inflammatory syndrome: a meta-analysis
Source: BMC Infect Dis. 2017 Apr 24;17:302. doi: 10.1186/s12879-017-2396-7 (PMC5404674; doi:10.1186/s12879-017-2396-7)
Supplement: Supplementary file 2 — Table of bias. Table of bias of neonatal and paediatric studies according to QUADAS-2. (PDF 84 kb) [file 12879_2017_2396_MOESM2_ESM.pdf]

Study	RISK OF BIAS				APPLICABILITY CONCERNS		
	**Patient Selection**	**Index Test**	**Reference Standard**	**Flow and Timing**	**Patient Selection**	**Index Test**	**Reference Standard**
**Neonatal**							
Adib 2012	😐	☺	☺	☺	☺	☺	☺
Bender 2008	☺	☺	☺	☺	☺	☺	☺
Bonac 2000	😐	☺	😐	☺	☺	☺	☺
Boo 2008	☺	☺	☺	☺	☺	☺	☺
Groselj- Grenc 2009	☺	☺	☹	☺	☺	☺	☺
Guibourdenche 2002	☹	☺	😐	☺	☺	☺	☺
Koskenvuo 2003	😐	☺	😐	☺	☺	☺	☺
Lopez Sastre 2006	☺	☺	☺	☺	☺	☺	☺
Naher 2011	☹	☺	☺	☺	☺	☺	☺
Resch 2003	☺	☺	☺	☹	☺	☺	☺
Sakha 2008	😐	☺	☺	☺	☺	☺	☺
Schlapbach 2013	☺	☺	☹	☺	☺	☺	☺
Vazzalwar 2004	☺	☺	☺	☺	☺	☺	☺
Zahedpasha 2009	😐	☺	☺	☺	☺	☺	☺
**Paediatric**							
Calò Carducci 2014	☺	☺	😐	☺	☺	☺	☺
Groselj- Grenc 2009	☺	☺	☹	☺	☺	☺	☺
Pourakbari 2010	😐	☺	☺	☺	☺	☺	😐
Simon 2008	☺	☺	☺	☺	☺	☺	☺
