# Supplementary material for: Accuracy of serum procalcitonin for the diagnosis of sepsis in neonates and children with systemic inflammatory syndrome: a meta-analysis
Source: BMC Infect Dis. 2017 Apr 24;17:302. doi: 10.1186/s12879-017-2396-7 (PMC5404674; doi:10.1186/s12879-017-2396-7)
Supplement: Supplementary file 3 — Table of included neonatal studies. CR, Chart review; DOR, diagnostic odds ratio; LR +, positive likelihood ratio; LR -, negative likelihood ratio; MC, Microbiologically confirmation; PCT, procalcitonin; SIRS, systemic inflammatory response syndrome; MC (Microbiologically confirmation). (PDF 130 kb) [file 12879_2017_2396_MOESM3_ESM.pdf]

Study	Early or Late Onset	n	Prevalence of sepsis (%)	Timing of test	PCT Cut-off (ng/mL)	Inclusion Criteria	Diagnosis of sepsis	Sensitivity	Specificity	LR+	LR-	DOR	Ln(DOR)	St error (ln(DOR))
Adib 2012	Early and Late	69	29	0h	1.15	Suspected sepsis	MC	0.700	0.400	1..2	0.75	1.6	0.44	0.57
Bender 2008	Early	123	24	0h	5.75	Suspected sepsis	MC or CR	0.690	0.670	2.1	0.46	4.5	1.51	0.46
					25			0.207	0.926	2.8	0.86	3.2	1.18	0.60
Bonac 2000	Early	58	15	0h	9.98	Suspected sepsis	MC or CR	0.520	0.910	5.8	0.53	11.0	2.39	0.83
				24h	13.03			0.590	0.820	3.3	0.50	6.6	1.88	0.77
				48h	3.07			0.500	1.000	2450.5	0.50	4900.0	8.50	10.02
Boo 2008	Early and Late	87	21	0h	0,5	Suspected Sepsis	MC	0.889	0.406	1.5	0.27	5.5	1.70	0.79
				12-24 h	2			0.889	0.652	2.6	0.17	15.0	2.71	0.79
				36-48 h	10			0.722	0.754	2.9	0.37	8.0	2.07	0.60
Groselj-Grenc 2009	Early and Late	46	63	0h	2.28	SIRS	MC or CR	0.824	0.483	1.6	0.37	4.4	1.47	0.74
				24h	5.55			0.313	1.000	906.6	0.69	1318.2	7.18	10.02
Guibourdenche 2002	Early	120	18	0-72h	2.5	Suspected sepsis	MC or CR	0.857	0.898	8.4	0.16	52.7	3.96	0.72
Koskenvuo 2003	Early	22	22	12 h	2	SIRS	MC or CR	0.998	0.176	1.2	0.01	107.1	4.67	10.03
Lopez Sastre 2006	Late	100	61	0 h	0.59	Suspected sepsis	MC	0.820	0.795	4.0	0.23	17.6	2.87	0.52
				12-24 h	1.34			0.869	0.718	3.1	0.18	16.9	2.83	0.52
				36-48 h	0.69			0.738	0.795	3.6	0.33	10.9	2.39	0.49
Naher 2011	Early and Late	50	80	0h	0.5	Suspected Sepsis	MC or CR	0.900	0.550	2.0	0.18	11.0	2.40	1.10
Resch 2003	Early	68	61	0h	2	Suspected Sepsis	MC or CR	0.830	0.610	2.1	0.28	7.6	2.03	0.57
					6			0.770	0.910	8.6	0.25	33.9	3.52	0.77
					14			0.630	1.000	1701.6	0.37	4597.3	8.43	10.01
Sakha 2008	Early and Late	117	23	-	2.5	Suspected sepsis	MC	0.667	0.500	1.3	0.67	2.0	0.69	0.46
Schlapbach 2013	Early	137	24.1	0 h	2	Suspected sepsis	MC or CR	0.879	0.510	1.8	0.24	7.5	2.02	0.57
Vazzalwar 2004	Late	51	35.3	0 h	0.5	Suspected sepsis	MC or CR	0.944	0.364	1.5	0.15	9.7	2.27	1.09
					1			0.778	0.636	2.1	0.35	6.1	1.81	0.67
Zahedpasha 2009	Early and Late	38	28.9	0 h	0.5	Suspected sepsis	MC or CR	0.999	0.259	1.3	0.00	385.0	5.95	10.01
					2			0.999	0.333	1.5	0.00	550.0	6.31	10.01
					10			0.909	0.852	6.1	0.11	57.5	4.05	1.18
