# Supplementary material for: Accuracy of serum procalcitonin for the diagnosis of sepsis in neonates and children with systemic inflammatory syndrome: a meta-analysis
Source: BMC Infect Dis. 2017 Apr 24;17:302. doi: 10.1186/s12879-017-2396-7 (PMC5404674; doi:10.1186/s12879-017-2396-7)

**PCT neon - cut-off < 2**

| Study | TP | FP | FN | TN | Cut-off | Timing | Onset | Sensitivity (95% CI) | Specificity (95% CI) |
|---|---|---|---|---|---|---|---|---|---|
| Zahedpasha 2009 | 11 | 20 | 0 | 7 | 0.5 | T0 | Early+Late | 1.00 [0.72, 1.00] | 0.26 [0.11, 0.46] |
| Naher 2011 | 9 | 18 | 1 | 22 | 0.5 | T0 | Early+Late | 0.90 [0.55, 1.00] | 0.55 [0.38, 0.71] |
| Boo 2008 | 16 | 41 | 2 | 28 | 0.5 | T0 | Early+Late | 0.89 [0.65, 0.99] | 0.41 [0.29, 0.53] |
| Adib 2012 | 14 | 29 | 6 | 20 | 1.15 | T0 | Early+Late | 0.70 [0.46, 0.88] | 0.41 [0.27, 0.56] |
| Lopez Sastre 2006 | 50 | 8 | 11 | 31 | 0.59 | T0 | Late | 0.82 [0.70, 0.91] | 0.79 [0.64, 0.91] |
| Vazzalwar 2004 | 14 | 12 | 4 | 21 | 1.0 | T0 | Late | 0.78 [0.52, 0.94] | 0.64 [0.45, 0.80] |

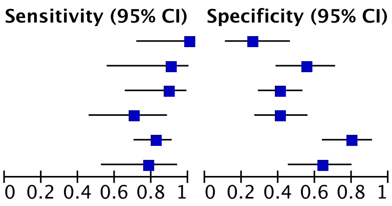


**PCT neon - cut-off = 2/2.5**

| Study | TP | FP | FN | TN | Cut-off | Timing | Onset | Sensitivity (95% CI) | Specificity (95% CI) |
|---|---|---|---|---|---|---|---|---|---|
| Resch 2003 | 34 | 10 | 7 | 17 | 2.0 | T0 | Early | 0.83 [0.68, 0.93] | 0.63 [0.42, 0.81] |
| Guibourdenche 2002 | 18 | 9 | 3 | 79 | 2.5 | T0 | Early | 0.86 [0.64, 0.97] | 0.90 [0.81, 0.95] |
| Koskenvuo 2003 | 5 | 14 | 0 | 3 | 2.0 | T12 | Early | 1.00 [0.48, 1.00] | 0.18 [0.04, 0.43] |
| Schlapbach 2013 | 29 | 51 | 4 | 53 | 2.0 | T24-72 | Early | 0.88 [0.72, 0.97] | 0.51 [0.41, 0.61] |
| Boo 2008 | 16 | 24 | 2 | 45 | 2.0 | T0 | Early+Late | 0.89 [0.65, 0.99] | 0.65 [0.53, 0.76] |
| Zahedpasha 2009 | 11 | 18 | 0 | 9 | 2.0 | T0 | Early+Late | 1.00 [0.72, 1.00] | 0.33 [0.17, 0.54] |
| Groselj-Grenc 2009 | 14 | 15 | 3 | 14 | 2.28 | T0 | Early+Late | 0.82 [0.57, 0.96] | 0.48 [0.29, 0.67] |
| Sakha 2008 | 18 | 45 | 9 | 45 | 2.5 | T0 | Early+Late | 0.67 [0.46, 0.83] | 0.50 [0.39, 0.61] |

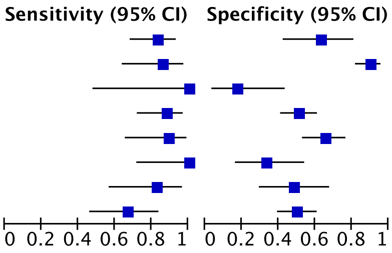


**PCT neon - cut-off > 2.5**

| Study | TP | FP | FN | TN | Cut-off | Timing | Onset | Sensitivity (95% CI) | Specificity (95% CI) |
|---|---|---|---|---|---|---|---|---|---|
| Bender 2008 | 20 | 31 | 9 | 63 | 5.75 | T0 | Early | 0.69 [0.49, 0.85] | 0.67 [0.57, 0.76] |
| Resch 2003 | 32 | 2 | 9 | 24 | 6.0 | T0 | Early | 0.78 [0.62, 0.89] | 0.92 [0.75, 0.99] |
| Bonac 2000 | 5 | 9 | 4 | 40 | 9.98 | T0 | Early | 0.56 [0.21, 0.86] | 0.82 [0.68, 0.91] |
| Zahedpasha 2009 | 10 | 4 | 1 | 23 | 10.0 | T0 | Early+Late | 0.91 [0.59, 1.00] | 0.85 [0.66, 0.96] |
| Boo 2008 | 13 | 17 | 5 | 52 | 10.0 | T0 | Early+Late | 0.72 [0.47, 0.90] | 0.75 [0.64, 0.85] |
| Groselj-Grenc 2009 | 5 | 0 | 11 | 29 | 5.55 | T24 | Early+Late | 0.31 [0.11, 0.59] | 1.00 [0.88, 1.00] |

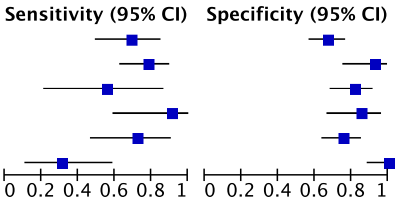

Supplement: Supplementary file 4 — Forest plot of studies on PCT for diagnosis of sepsis in neonatal age. The forest plot represents in each study the sensitivity and the specificity of PCT, together with the 95% CI for diagnosis of sepsis in neonatal age stratified according cut-off subgroup. (CI, confidence interval; FP, false positive; FN, false negative; PCT, procalcitonin; TP, true positive; TN, true negative). (PDF 743 kb) [file 12879_2017_2396_MOESM4_ESM.pdf]
