# Supplementary figures and images for: Accuracy of serum procalcitonin for the diagnosis of sepsis in neonates and children with systemic inflammatory syndrome: a meta-analysis
Source: BMC Infect Dis. 2017 Apr 24;17:302. doi: 10.1186/s12879-017-2396-7 (PMC5404674; doi:10.1186/s12879-017-2396-7)

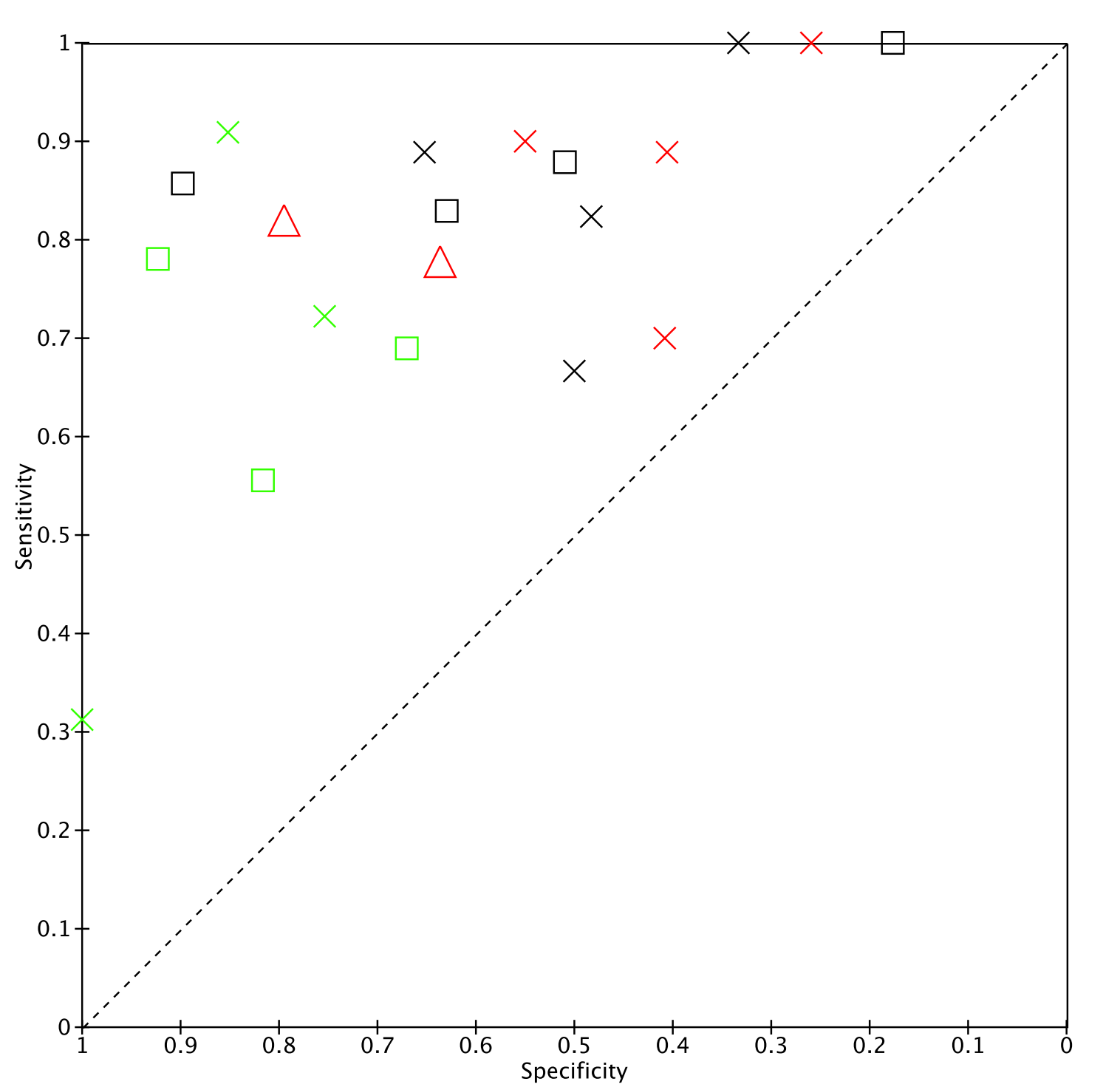

Supplement: Supplementary file 5 — Representation in the ROC space of neonatal studies divided in EOS and LOS. Representation in the ROC space of studies on PCT for diagnosis of sepsis in neonatal age, divided by cut-off subgroup and EOS/LOS. (ROC, receiver operating characteristic). (PDF 46 kb) [file 12879_2017_2396_MOESM5_ESM.pdf]

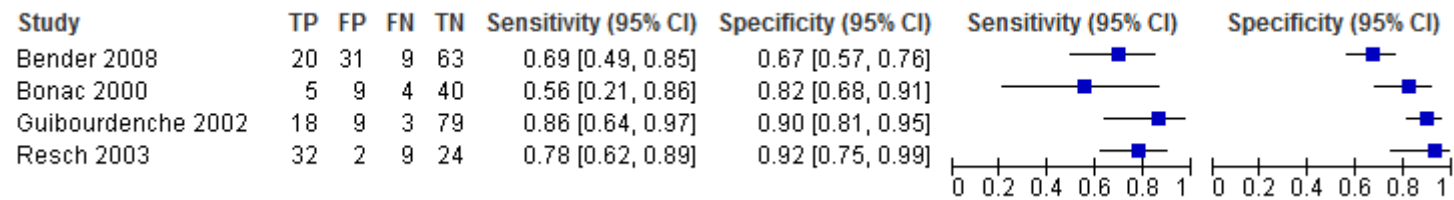

| Cut-off | Study               | Summary statistics     |
|---------|---------------------|------------------------|
| >2.5    | Bender (5.75)       |                        |
|         | Bonac (9.98)        | SE = 0.75 (0.64; 0.84) |
|         | Guibourdenche (2.5) | SP = 0.83 (0.71; 0.91) |
|         | Resch (6)           |                        |

Supplement: Supplementary file 6 — Forest plot and summary statistics of studies on PCT for diagnosis of EOS. The forest plot represents in each study the sensitivity and the specificity of PCT, together with the 95% CI for diagnosis of EOS. (CI, confidence interval; FP, false positive; FN, false negative; PCT, procalcitonin; TP, true positive; TN, true negative). (PDF 323 kb) [file 12879_2017_2396_MOESM6_ESM.pdf]

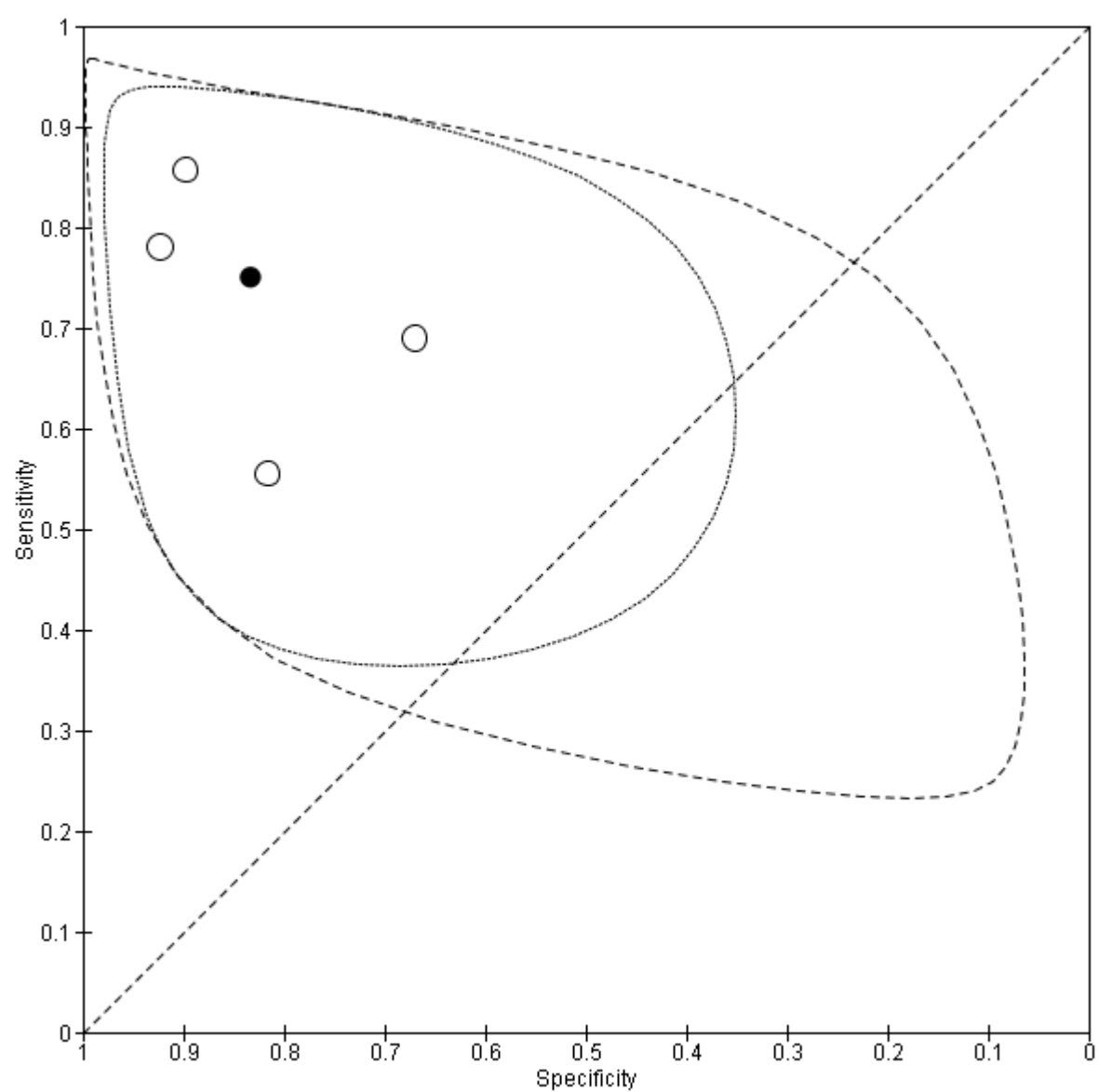

Supplement: Supplementary file 7 — Representation in the ROC space of studies in EOS. Representation in the ROC space of studies on PCT for diagnosis of EOS. (ROC, receiver operating characteristic). (PDF 157 kb) [file 12879_2017_2396_MOESM7_ESM.pdf]

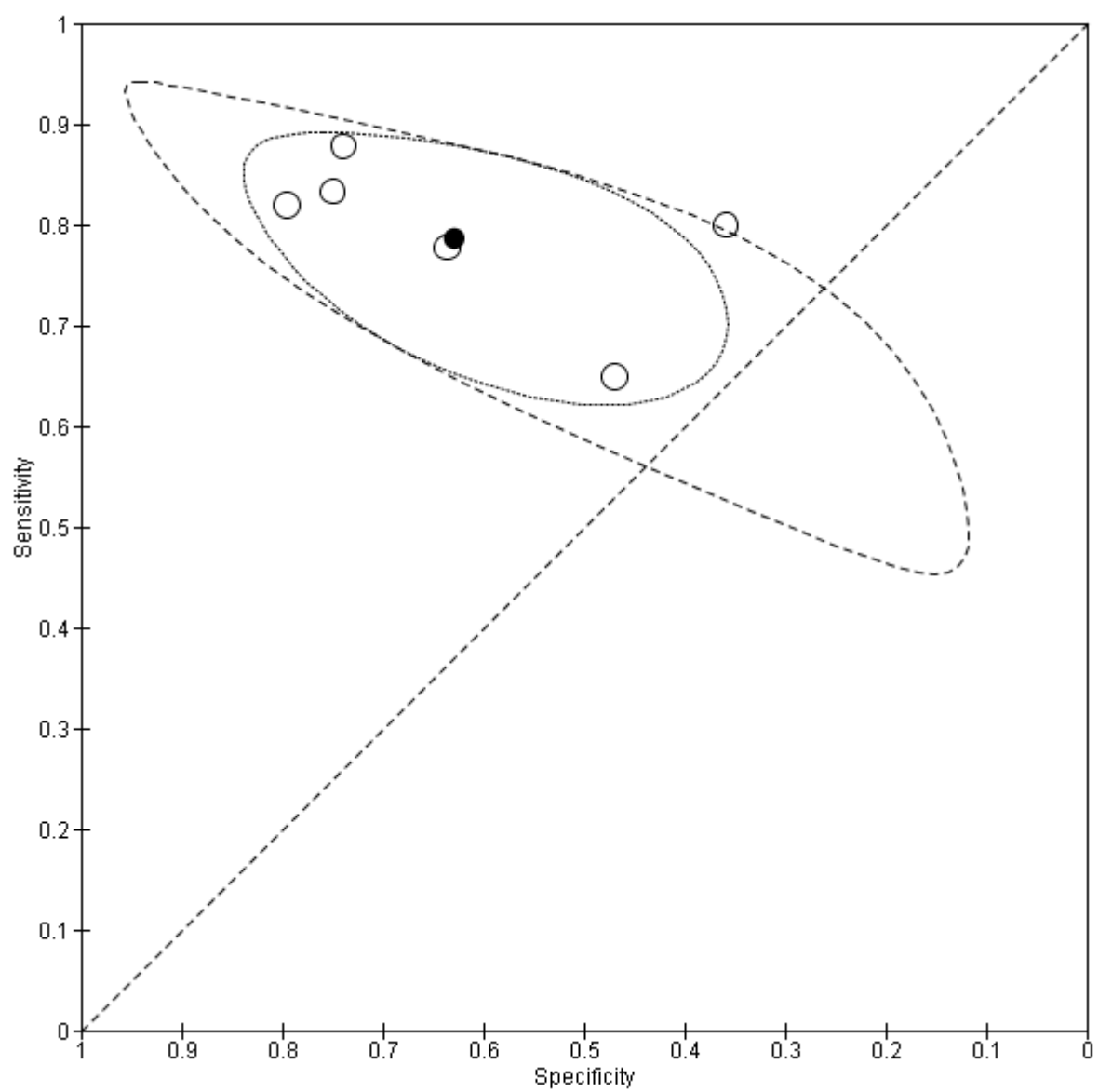

Supplement: Supplementary file 11 — Representation in the ROC space of studies in LOS and paediatric sepsis. Representation in the ROC space of studies on PCT for diagnosis in LOS and paediatric sepsis. (PDF 154 kb) [file 12879_2017_2396_MOESM11_ESM.pdf]
