# Supplementary material for: Accuracy of serum procalcitonin for the diagnosis of sepsis in neonates and children with systemic inflammatory syndrome: a meta-analysis
Source: BMC Infect Dis. 2017 Apr 24;17:302. doi: 10.1186/s12879-017-2396-7 (PMC5404674; doi:10.1186/s12879-017-2396-7)
Supplement: Supplementary file 8 — Table of included paediatric studies. (DOR, diagnostic odds ratio; LR+, positive likelihood ratio; LR-, negative likelihood ratio; PCT, procalcitonin; SIRS, systemic inflammatory response syndrome; MC, Microbiologically confirmation; CR Chart Review). (PDF 19 kb) [file 12879_2017_2396_MOESM8_ESM.pdf]

Additional file 8. Table of included pediatric studies.

Study	n	Prevalence of sepsis	Timing of test	PCT Cut-off (ng/mL)	Inclusion Criteria	Outcome Diagnosis	Sensitivity %	Specificity %	LR +	LR -	DOR	Ln(DOR)	St error (ln(DOR))
Calò Carducci 2014	64	64	0h	0.55	SIRS	MC or CR	0.878	0.739	3.4	0.16	20.4	3.02	0.67
Groselj-Grenc 2009	36	67	0h	0.28	SIRS	CR	0.833	0.750	3.3	0.22	15.0	2.71	0.86
			24h	0.65			0.810	0.880	6.8	0.22	31.3	3.44	1.04
Pourakbari 2010	158	79	0h	0.5	SIRS	MC	0.800	0.359	1.2	0.56	2.2	0.81	0.60
				2			0.680	0.744	2.7	0.43	6.2	1.82	0.56
				10			0.520	0.821	2.9	0.59	5.0	1.60	0.58
Simon 2008	64	39	24h	0.5	SIRS	CR	0.649	0.469	1.2	0.75	1.6	0.49	0.34
				2.5			0.439	0.796	2.1	0.71	3.0	1.11	0.37
				5			0.298	0.888	2.7	0.79	3.4	1.21	0.43

CR, Chart review; DOR, diagnostic odds ratio; LR +, positive likelihood ratio; LR -, negative likelihood ratio; MC, Microbiologically confirmation; PCT, procalcitonin; SIRS, systemic inflammatory response syndrome.
