# Supplementary material for: Accuracy of serum procalcitonin for the diagnosis of sepsis in neonates and children with systemic inflammatory syndrome: a meta-analysis
Source: BMC Infect Dis. 2017 Apr 24;17:302. doi: 10.1186/s12879-017-2396-7 (PMC5404674; doi:10.1186/s12879-017-2396-7)

PCT ped - cut-off < 2

Study	TP	FP	FN	TN	Cut-off	Sensitivity (95% CI)	Specificity (95% CI)
Calo' Carducci 2014	36	6	5	17	0.55	0.88 [0.74, 0.96]	0.74 [0.52, 0.90]
Groselj-Grenc 2009	20	3	4	9	0.28	0.83 [0.63, 0.95]	0.75 [0.43, 0.95]
Pourakbari 2010	37	52	20	46	0.5	0.65 [0.51, 0.77]	0.47 [0.37, 0.57]
Simon 2008	20	25	5	14	0.5	0.80 [0.59, 0.93]	0.36 [0.21, 0.53]

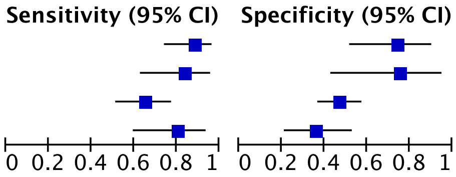

PCT ped - cut-off = 2/2.5

Study	TP	FP	FN	TN	Cut-off	Sensitivity (95% CI)	Specificity (95% CI)
Pourakbari 2010	25	20	32	78	2.0	0.44 [0.31, 0.58]	0.80 [0.70, 0.87]
Simon 2008	17	10	8	29	2.5	0.68 [0.46, 0.85]	0.74 [0.58, 0.87]

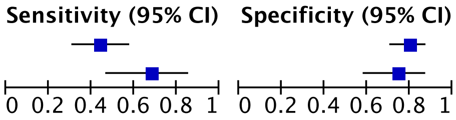

PCT test ped - cut-off > 2.5

Study	TP	FP	FN	TN	Cut-off	Sensitivity (95% CI)	Specificity (95% CI)
Pourakbari 2010	17	11	40	87	10.0	0.30 [0.18, 0.43]	0.89 [0.81, 0.94]
Simon 2008	13	7	12	32	5.0	0.52 [0.31, 0.72]	0.82 [0.66, 0.92]

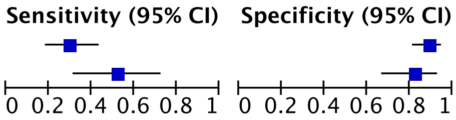

Supplement: Supplementary file 9 — Forest plot of studies on PCT for diagnosis of sepsis in paediatric age. The forest plot represents in each study the sensitivity and the specificity of PCT, together with the 95% CI for diagnosis of sepsis in paediatric age stratified according cut-off subgroup. (PDF 377 kb) [file 12879_2017_2396_MOESM9_ESM.pdf]
