# Supplementary material for: Accuracy of serum procalcitonin for the diagnosis of sepsis in neonates and children with systemic inflammatory syndrome: a meta-analysis
Source: BMC Infect Dis. 2017 Apr 24;17:302. doi: 10.1186/s12879-017-2396-7 (PMC5404674; doi:10.1186/s12879-017-2396-7)
Supplement: Supplementary file 10 — Forest plot and summary statistics of studies on PCT for diagnosis of LOS and paediatric sepsis. The forest plot represents in each study the sensitivity and the specificity of PCT, together with the 95% CI for diagnosis of LOS and paediatric sepsis. (CI, confidence interval; FP, false positive; FN, false negative; PCT, procalcitonin; TP, true positive; TN, true negative). (PDF 337 kb) [file 12879_2017_2396_MOESM10_ESM.pdf]

Study	TP	FP	FN	TN	Sensitivity (95% CI)	Specificity (95% CI)
Calo' Carducci 2014	36	6	5	17	0.88 [0.74, 0.96]	0.74 [0.52, 0.90]
Groselj-Grenc 2009	20	3	4	9	0.83 [0.63, 0.95]	0.75 [0.43, 0.95]
Lopez Sastre 2006	50	8	11	31	0.82 [0.70, 0.91]	0.79 [0.64, 0.91]
Pourakbari 2010	37	52	20	46	0.65 [0.51, 0.77]	0.47 [0.37, 0.57]
Simon 2008	20	25	5	14	0.80 [0.59, 0.93]	0.36 [0.21, 0.53]
Vazzalwar 2004	14	12	4	21	0.78 [0.52, 0.94]	0.64 [0.45, 0.80]

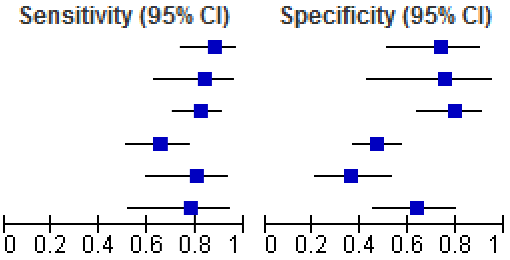

PCT Cut-off	Study	Summary statistics
**<2.0**	Groselj-Grenc (0.28)<br>Pourabaki (0.5)<br>Simon (0.5)<br>Calò Carducci (0.55)<br>Lopez Sastre 2006 (0.59)<br>Vazzalwar 2004 (1)	**SE = 0.79 (0.71; 0.85)**<br>**SP = 0.63 (0.48; 0.75)**
